# Acrokeratosis verruciformis of Hopf: Genetic, clinicopathologic and therapeutic features (Review)

**DOI:** 10.3892/mi.2026.321

**Published:** 2026-05-12

**Authors:** Konstantinos-Antonios Kostopoulos-Kanitakis, Jean Kanitakis

**Affiliations:** 1 Department of Dermatology, Centre Hospitalier Lyon Sud, Hospices Civils de Lyon, 69152 Pierre-Bénite, France; 2 Department of Dermatology/CliMA, Ed. Herriot Hospital (Pav. R), Hospices Civils de Lyon, 69437 Lyon cedex 03, France

**Keywords:** acrokeratosis verruciformis, Hopf, Darier disease, ATP2A2

## Abstract

Acrokeratosis verruciformis of Hopf (AVH) is a rare genodermatosis belonging to the group of epidermal differentiation diseases. It is due to the heterozygous missense mutation p.(Pro602Leu) in the ATPase sarcoplasmic/endoplasmic reticulum Ca^2+^ transporting 2 (*ATP2A2*) gene, which is also involved in Darier disease (DD), and is therefore considered an allelic form of DD. AVH is usually transmitted as an autosomal dominant trait with variable penetrance, although several sporadic cases have been reported. The disease manifests clinically with flat, wart-like papules with a verrucous surface that develop predominantly on the dorsum of the hands and feet, and appear early in life or, in sporadic cases, in adulthood. The lesions exhibit characteristic microscopic changes (verrucous epidermal hyperplasia with a ‘church-spire-like’ surface), which allow differentiation from DD. The course of the disease is chronic and the outcome as a rule benign, although rare cases of malignant transformation have been reported. Treatment is not necessary; it can be attempted at the request of the patient, knowing that no standardized treatment exists. Treatments that have been tested include topical agents (namely keratolytics, retinoids and cryotherapy) and systemic retinoids; however, the results are often modest. The present brief narrative review aimed to summarize the etiopathogenic, clinical, histopathological and therapeutic features of AVH.

## 1. Introduction

Acrokeratosis verruciformis of Hopf (AVH; OMIM #101900), first described in 1931 by Gustav Hopf (1), is a genodermatosis due to a trouble of epidermal differentiation, usually transmitted as an autosomal dominant trait with variable penetrance. It has characteristic clinicopathological features, although these partly overlap with those of Darier disease (DD); thus, the autonomy of AVH as a separate condition has been questioned in the past. The present study provides a brief narrative review of the etiopathogenic, clinical, histopathological and therapeutic features of AVH, based on a review of the literature following a PubMed search using as key words ‘acrokeratosis verruciformis’ and ‘Hopf’.

## 2. Etiopathogenesis

The existence of AVH as a separate entity has been questioned in the past, as lesions almost identical with those of AVH can be observed in patients with DD or their first-degree relatives (2-4). Furthermore, the two diseases may reportedly co-exist in the same patients (4-10), and histological findings similar to those of DD have occasionally been reported in AVH lesions (11). The confusion between the two entities has also been maintained by cases published as AVH, which in fact were cases of DD (12). It is now known that AVH is linked to pathogenic variants affecting the same gene as that mutated in DD, i.e. *ATPase sarcoplasmic/endoplasmic reticulum Ca*^2+^ *transporting 2* (*ATP2A2*) (13). This gene is localized to the chromosomal locus 12q23-q24; it encodes the calcium pump SERCA2, which is present in the membrane of the endo-sarcoplasmic reticulum and plays a key role in calcium transport, and indirectly in the intercellular adhesion of keratinocytes. However, the mutations causing the two diseases differ: whereas in DD a broad spectrum of mutations (missense or nonsense) has been identified (3,13), AVH is associated with the specific heterozygous missense mutation p.(Pro602Leu), present in exon 14 of the *ATP2A2* gene (11,14). This mutation completely abolishes the calcium transport activity of the protein. AVH is therefore currently considered an allelic form of DD (3,14). Of note, however, the p.(Pro602Leu) mutation has not been found in all patients with AVH (namely in Chinese patients), a fact suggesting that other genes could be involved in AVH (15). Moreover, several sporadic, non-familial AVH cases have been reported (7,16-19); these may reflect *de novo* mutations or reduced penetrance, particularly when there is no family history of a similar condition.

Apart from DD, AVH has also been reported in association with Hailey-Hailey disease (20), congenital poikiloderma (21), hereditary epidermolysis bullosa (22), sebocystomatosis, hypertrophic lichen planus (23), multiple keratoacanthomas (24), basal cell nevus syndrome (25), psoriasis (26), diabetes mellitus (27,28), hypertension, hypercholesterolemia, gastroesophagal reflux (27) and dilated cardiomyopathy (29). The significance of these associations is not known, although they could be coincidental. Human papillomavirus (HPV)17 has been detected in one case of AVH (9); however, search for HPV was negative in two other cases (19,30), casting shadow on the role of HPV in the development of the lesions.

## 3. Epidemiology

AVH is a rare condition; however, its precise incidence remains unknown. According to a previous study, the estimated frequency is 1 case per 41,000 dermatological consultations, i.e. half that of DD (31). Both sexes are affected, with no obvious sex predilection. AVH has been reported in patients from Asia, Europe, America and Australia. Even though many of the reported cases were sporadic (7,16-19,32-34), the disease is usually inherited, transmitted as an autosomal dominant trait with variable penetrance (11,13,15). Patients should therefore be warned that their offspring could develop the disease.

## 4. Clinical features

The cutaneous lesions of AVH usually appear during childhood or adolescence. They may be present at birth, particularly in familial cases, or may appear later, up to the 6th life decade, mostly in sporadic cases (33). The lesions present clinically as small, flat papules a few millimeters in size, resembling flat warts. They are flesh-colored or slightly pigmented and have a verrucous surface. They are sometimes more easily felt by palpation than are visible. They are asymptomatic or, rarely, slightly pruritic (8,27). They develop predominantly on the dorsum of the hands, fingers and feet. In sporadic cases, the lesions may be more diffuse, affecting the forearms, legs, elbows, knees, large body folds (9), the face (4) and the genitalia (35). A blaschkoid distribution was reported in one case (35). Giant plantar lesions that hinder walking have been described (28). Punctiform depressions (‘pits’) of the palatine mucosa were present in one patient (4). Dermatoscopy may support the diagnosis, even though the dermatoscopic findings are not entirely specific; they include a homogeneous whitish aspect, with occasional dot-like vessels (36,37). In patients with dark skin types, the lesions additionally exhibit whitish scales (38) and, occasionally, pigmented cobblestone areas (39). Punctiform hyperkeratotic lesions of the palms may be present (40). Nail lesions are not rare; they include pachyonychia, leukonychia, longitudinal depressions, subungual hemorrhages, distal onychoschizis, ‘V’ notches of the free edge and subungual hyperkeratosis (4,18,40,41), adding resemblance with DD. However, contrary to the latter, seborrheic body zones (such as the face, scalp and trunk) are usually spared in AVH (31). The lesions may darken in summer (15). Of note, two cases of squamous cell carcinoma that developed on long-standing AVH lesions have been reported (31,42).

## 5. Histological features

Routine histological examination of AVH reveals a well-circumscribed epidermal hyperplasia, consisting of orthokeratotic hyperkeratosis, acanthosis and moderate papillomatosis, often with hypergranulosis. The surface of the epidermis usually presents a characteristic verrucous, church-spire-like appearance. Spongiosis (7) and apoptotic keratinocytes (18) have been reported rarely. The dermis is most often normal but may rarely contain a sparse perivascular lymphocytic infiltrate (19,20,43). The presence of suprabasal clefts and ‘corps ronds’ in AVH is a matter of controversy in the literature; although the absence of these findings has been considered as a feature allowing differentiation of AVH from DD, some cases diagnosed as AVH have disclosed suprabasal acantholytic clefts and cells with a dyskeratotic tendency (11,44), highlighting the overlap between the two diseases. However, when present, acantholysis and dyskeratosis are typically minimal in AVH.

## 6. Diagnosis

The diagnosis of AVH relies mainly on clinical and histological examination. Conditions that may clinically mimic AVH include flat warts, epidermodysplasia verruciformis, stucco-keratoses and DD (5,19). Dermatoscopic examination can aid in the diagnosis, allowing namely to differentiate AVH from lichen planus, flat warts and epidermodysplasia verruciformis (43). Acral papules similar to those of AVH, although generally more keratotic, are present in another genodermatosis, Cowden syndrome (OMIM #158350), which is due to pathogenic variants of the *PTEN* gene. Contrary to AVH, this syndrome also manifests with multiple facial trichilemmomas and mucous membrane lesions, hamartomas and visceral cancers. Since the clinicopathologic features of AVH are not entirely specific, the diagnosis should be confirmed, whenever possible, by genetic analysis, specifically by demonstrating the p.(Pro602Leu) mutation in the *ATP2A2* gene. Of note, however, the majority of patients with AVH reported thus far in the literature, even recently, were not genetically examined, a fact that renders the diagnosis unconfirmed. In any case, when managing a patient with suspected AVH, DD should be ruled out by a thorough examination of the whole skin.

## 7. Course - prognosis

The course of AVH is chronic. The lesions persist indefinitely and do not regress spontaneously. AVH is, in the vast majority of cases, a benign condition, causing merely an aesthetic burden. Although rare, transformation into squamous cell carcinoma has been reported (31,42); therefore, longitudinal clinical follow-up of the patients is advised.

## 8. Treatment

Since AVH is a benign condition, treatment is not required, but may be initiated at the request of the patients, who may be motivated for aesthetic reasons. Due to the relatively limited number of cases reported until now, no randomized clinical trials have been performed in AVH. The treatments that have been tested are purely symptomatic, directed toward symptom control and cosmetic improvement. They include destructive methods used for warts, such as cryotherapy and topical medications (mainly keratolytics and retinoids). Topical tretinoin has shown limited efficacy (2,4,27). A partial response was obtained with tazarotene in one patient (7). The lesions may, however, be refractory to topical treatments, as shown by a case where several topical agents (keratolytics with urea and salicylic acid 40%, clobetasol 0.05%, tretinoin 0.1%, pumice stone abrasion) proved ineffective (27). Systemic retinoids yield variable results: acitretin (0.5-0.7 mg/day) has proven effective in 2 cases (24,45); however, the lesions may reappear following the discontinuation of treatment (45). In another patient treated with oral acitretin (12.5 mg/day), the results were deemed ‘satisfactory’ (43). One patient with giant lesions was treated with oral acitretin (25 mg/day) in combination with weekly cryotherapy sessions (two freeze/thaw cycles of 5-10 sec each per lesion); significant improvement (lesion flattening) was observed after one month, particularly on plantar lesions (28). Etretinate proved ineffective in two patients (16,17). Oral alitretinoin (10 mg/day) was tried in two patients; modest results were obtained in one case (33) (the result was not mentioned in the second case) (41). Three patients were treated with oral isotretinoin (20-40 mg/day) in association with local treatments (keratolytics, moisturizers, tretinoin 0.025%, topical corticoids, cryotherapy) with favorable results (8,46).

## 9. Conclusion

Acrokeratosis verruciformis of Hopf is a rare epidermal differentiation disorder, regarded as an allelic form of Darier disease, inherited as an autosomal dominant trait with variable penetrance. It is linked to a specific mutation p.(Pro602Leu) localized in exon 14 of the ATP2A2 gene, and manifests with verrucous papules predominantly affecting the dorsum of the hands and feet. It has a protracted, as a rule benign, course, even though exceptional cases of transformation into cutaneous squamous cell carcinoma have been reported. Treatments that have been tried with variable success are based mainly on topical destructive methods (cryotherapy, keratolytics) in association with systemic retinoids. Description of additional cases will hopefully provide further insights into this rare dermatosis.

## Data Availability

Not applicable.
